# Primary Extrapulmonary Rifampicin Mono-Resistant Tuberculosis of the Knee in an Indian Female Without Pulmonary Involvement: The World's First Case

**DOI:** 10.7759/cureus.43784

**Published:** 2023-08-20

**Authors:** Sankalp Yadav

**Affiliations:** 1 Medicine, Shri Madan Lal Khurana Chest Clinic, Moti Nagar, New Delhi, IND

**Keywords:** rifampicin mono-resistance tuberculosis, mtb (mycobacterium tuberculosis), tuberculosis, cbnaat/ xpert/ rif assay, knee joint

## Abstract

Extrapulmonary tuberculosis presentations are comparatively less frequent. Isolated involvement of the knee in the absence of pulmonary focus is extremely rare. Further, primary extrapulmonary rifampicin mono-resistant tuberculosis of the knee without pulmonary involvement is never reported in females. A case of a 26-year-old female is presented who came with complaints of pain, swelling, and discharging sinuses from her left knee. In the absence of a history of tuberculosis or trauma, the diagnosis was challenging. However, she was diagnosed based on the reports of cartridge-based nucleic acid amplification tests and clinical correlation with radiometric techniques and initiated anti-tuberculous treatment according to her weight per the national guidelines present in 2018.

## Introduction

Tuberculosis is a serious public health issue in developing countries [1]. The disease is an outcome of infection by acid-fast bacilli, *Mycobacterium tuberculosis*, belonging to the family Mycobacteriacae [2]. The data from the latest reports from India mentions that in the year 2021, the estimated incidence of multidrug-resistant or rifampicin mono-resistant patients will be 119,000 (93,000-145,000) [3]. Besides, there was an upside of 32% in the number of multidrug-resistant and rifampicin mono-resistant cases detected in 2022 compared to 2021 [3].

Rifampicin mono-resistant tuberculosis is a type of tuberculosis where the bacteria are resistant to rifampicin [3]. There is a rapid rise in the incidence of drug-resistant tuberculosis across the globe [3]. Often, the primary focus is on the lungs [4]. But sporadic cases of hematogenous, lymphatic spread, or local development of disease at extrapulmonary sites are available in the literature [4].

Herein, a case of a 26-year-old Indian female is presented who presented with swelling and discharging sinuses from her left knee. She was diagnosed after a detailed clinical, radiometric, and laboratory workup and initiated anti-tubercular treatment.

## Case presentation

In 2018, a 26-year-old Indian, unmarried, non-diabetic female reported to the outpatient department with complaints of pain, swelling, and discharging sinuses in her left knee for 18 months. The pain was sudden in onset, continuous, non-radiating, aggravated on walking (with a visible limp), and relieved (for two to four hours) by taking over-the-counter drugs. The swelling was insidious in its onset and has increased over the past month. There were two discharging sinuses over the left knee joint with purulent discharge for twenty days. There was no fever, cough, or history of weight loss.

There was no history of trauma or falls. Further, she was a student with no history of tuberculosis or any close contacts. She was a teetotaler and never smoked. Furthermore, there was no history of migration, imprisonment, or stays at night shelters or relief camps.

A general examination was suggestive of a medium-built female with a body mass index of 18.4 g/m2. She was hemodynamically stable. Local examination of the left knee revealed a swollen knee joint with two discharging sinuses about 2x2 cm and 1x2 cm in size with a yellow-colored, non-fowl-smelling discharge. There was a restriction of movement at the left knee joint with a positive patellar tap test. The local skin was erythematous with a raised temperature but no dilated veins. The right knee joint was normal. And there was no icterus, edema, lymphadenopathy, clubbing, cyanosis, or pallor. The vascular, pulmonary, abdominal, and neurological examinations were unremarkable.

**Figure 1:**
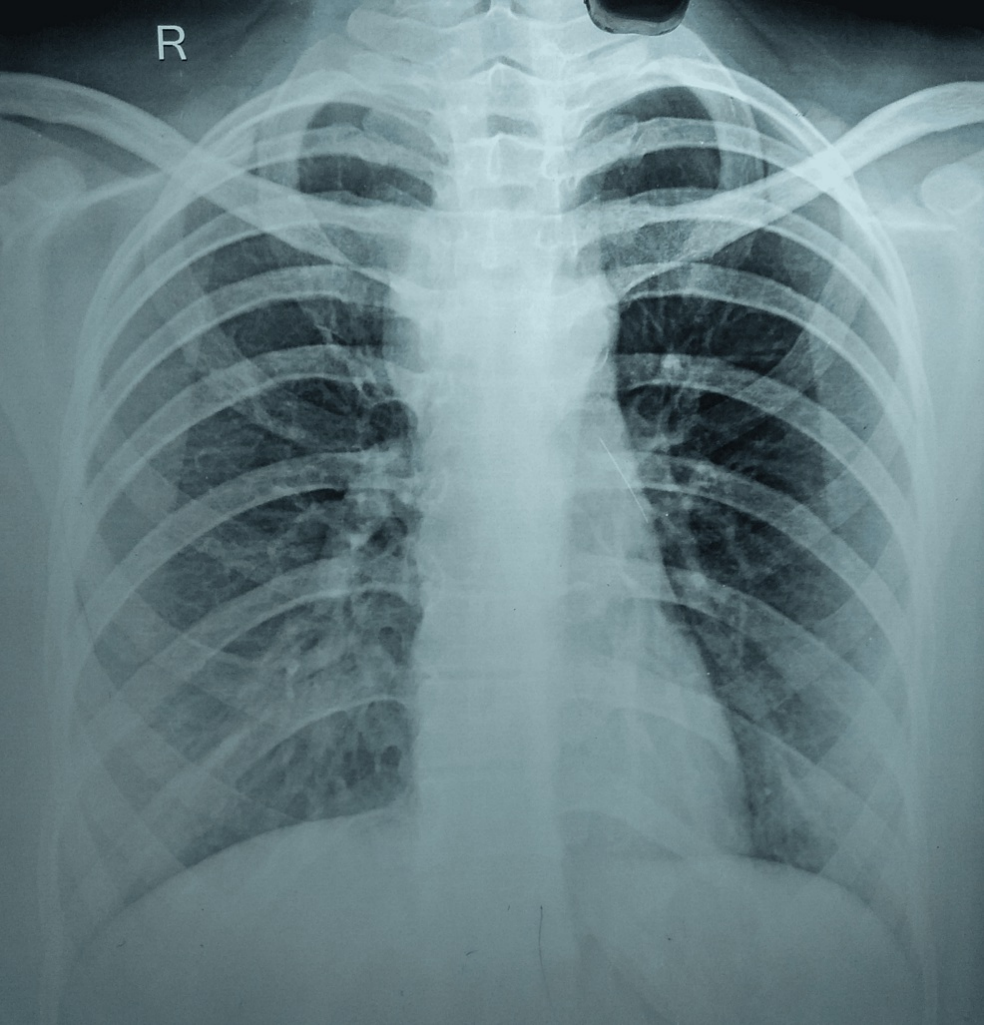
A plain radiograph of the chest not suggestive of pulmonary tuberculosis

Assuming she was a case of pyogenic abscess with differentials such as tuberculous osteomyelitis, bone tumor, and fungal osteomyelitis, a detailed lab and radiometric workup was planned. She underwent a routine blood workup with induced sputum smear microscopy and cartridge-based nucleic acid amplification tests. Her blood investigations were remarkable for a raised erythrocyte sedimentation rate of 51 mm in the first hour with a hemoglobin of 11.2 g/dL. HIV (I and II), hepatitis panel (A, B, and C), and rheumatoid factor were negative, but her Mantoux test was strongly positive (30 mm). A plain radiograph of the chest was not suggestive of pulmonary involvement (Figure 1).

**Figure 2:**
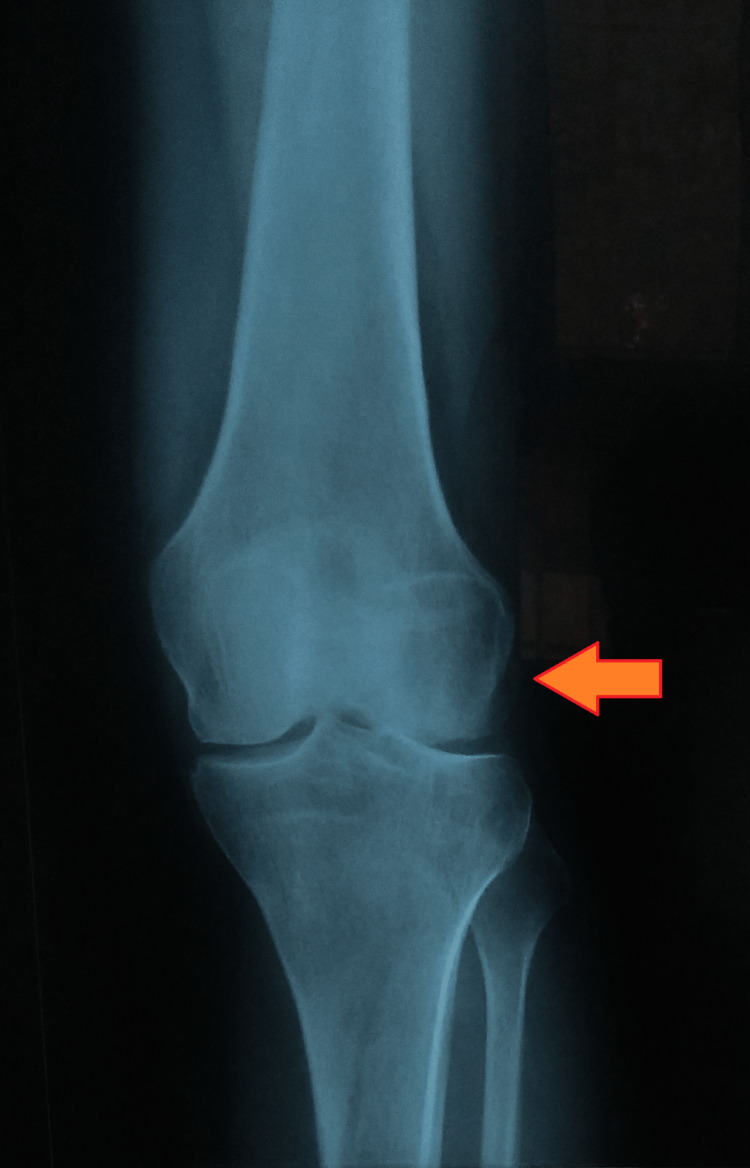
Plain radiograph of the left knee suggestive of bone demineralization of the lateral femoral condyle

A plain radiograph of the left knee was suggestive of bone demineralization of the lateral femoral condyle (Figure 2).

**Figure 3:**
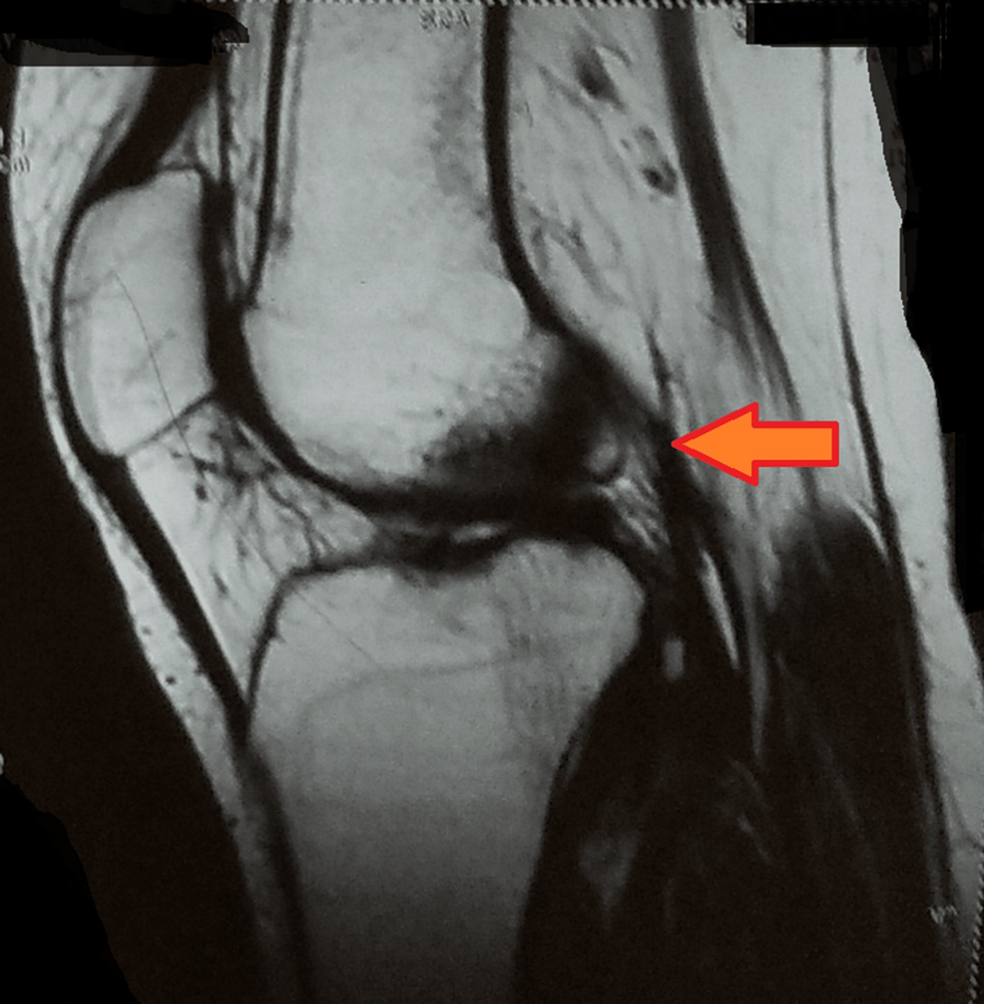
Magnetic resonance imaging- left knee joint Arrow showing hyperintensity in the lateral condyle of the femur and tibia

A magnetic resonance imaging of the left knee joint was suggestive of a grade-I meniscal signal in the anterior horn of the lateral meniscus with intra-substance strain seen along the anterior cruciate ligament, near its tibial attachment, with spared continuity. Minimal fluid was seen in the left knee joint, with hyperintensity in the lateral condyle of the femur and tibia (Figure 3).

A wound debridement with an open biopsy was done with the drainage of 20 ml of yellow-colored pus. Histopathology was suggestive of tuberculosis, with epitheloid granulomas in a necrotic background, occasional Langhans giant cells, and lymphocytic infiltrates. The smear of pus for acid-fast bacilli was negative on Ziehl-Neelsen staining. However, a cartridge-based nucleic acid amplification test detected *Mycobacterium tuberculosis* (low) with resistance to rifampicin.

**Figure 4:**
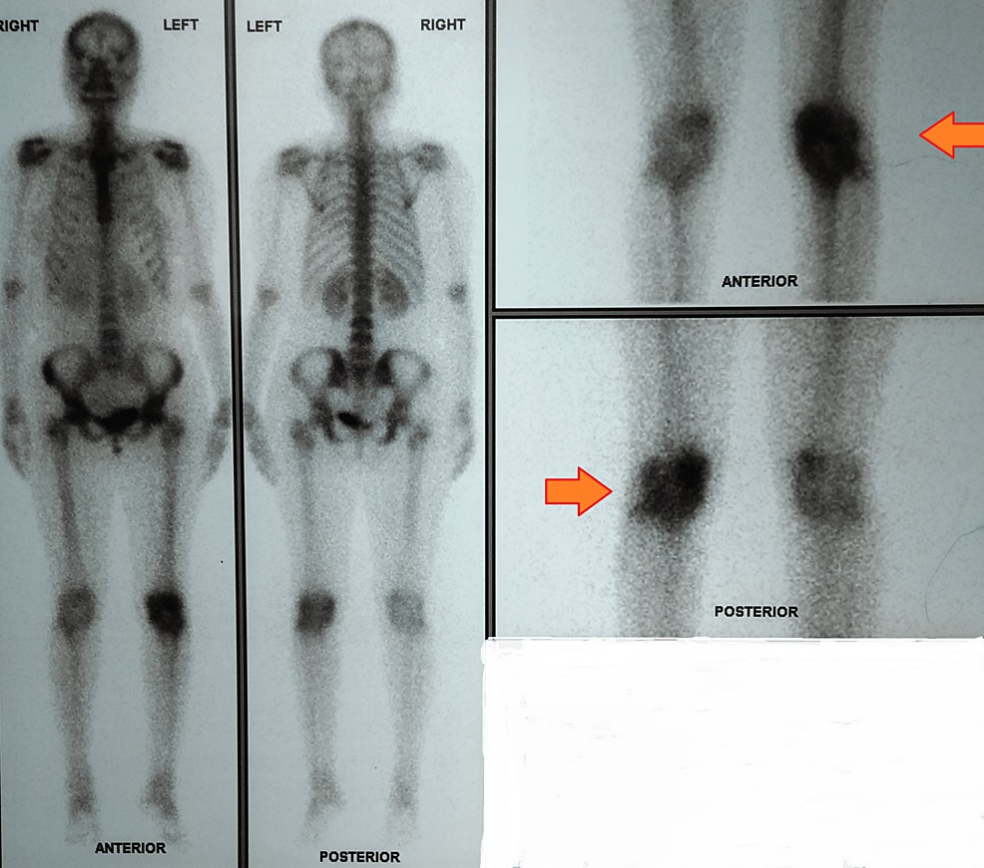
Positron emission tomography scan of the patient Arrows remarkable for increased osteoblastic activity involving the left knee with increased flow and blood pooling

The line-probe assay and culture of the pus were negative. Advanced radiometric investigations like positron emission tomography scans were remarkable for increased osteoblastic activity involving the left knee with increased flow and blood pooling consistent with infective pathology (Figure 4).

A final diagnosis of primary extrapulmonary rifampicin mono-resistant tuberculosis of the left knee joint without pulmonary involvement was made, and it was planned for a pretreatment evaluation to initiate the conventional regimen per the national guidelines [5]. As her pretreatment evaluation was unremarkable for any major issues, she was put on a conventional regimen (Table 1).

**Table 1:** Anti-tubercular chemotherapy conventional regimen per her weight IM: intramuscular; OD: once daily

Drug	Route of administration	Dose	Duration
Kanamycin (15mg/kg)	Intramuscular	750 mg X IM	6 months
Ethionamide (15 mg/kg)	Per oral	750 mg X OD	24 months
Levofloxacin (15 mg/kg)	Per oral	1000 mg X OD	24 months
Cycloserine (10 mg/kg)	Per oral	500 mg X OD	24 months
Ethambutol (25 mg/kg)	Per oral	1200 mg X OD	24 months
Pyrazinamide (25 mg/kg)	Per oral	1250 mg X OD	24 months
Pyridoxine (1 mg/kg)	Per oral	100 mg X OD	24 months

**Figure 5:**
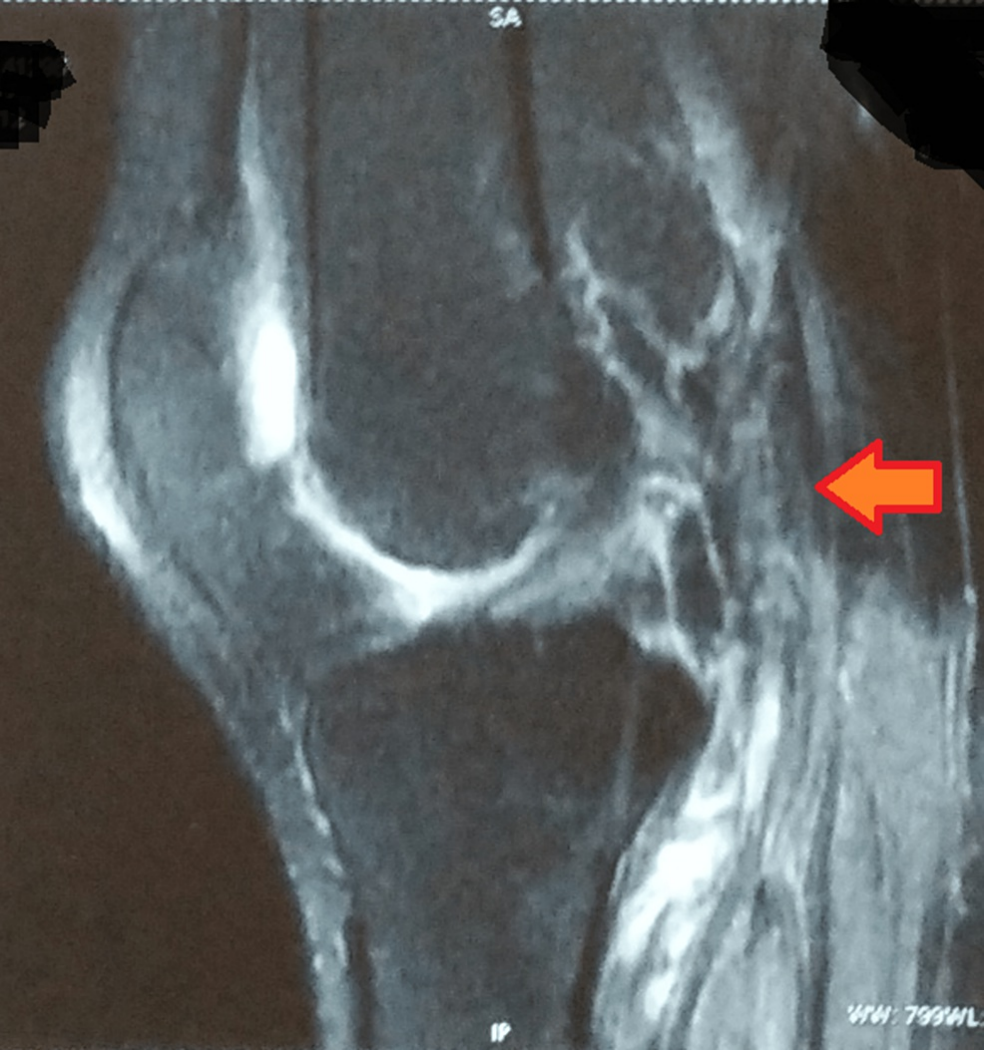
Magnetic resonance imaging without contrast-left knee joint at the six-month follow-up Arrow suggestive of joint involvement

She tolerated her anti-tubercular medicines well, with no major adverse drug reactions. There was a reduction in pain and swelling, and a six-month follow-up magnetic resonance imaging of the left knee joint was suggestive of articular erosions involving the trochlear surface of the femur with thinning of articular cartilage in the medial and lateral tibio-femoral compartments. There were diffuse fatty marrow changes in the bones, suggesting osteopenia or osteoporosis. There was a mild increase in the joint fluid with the intra-substance, which increased signal intensity in the body of the lateral meniscus. Mild tendinosis is seen in the patellar and quadriceps tendons. There was edema in the popliteus muscle and posterior intermuscular fat planes. A partially fibrosed inflammatory thickening was seen behind the lateral tibial condyle, reaching up to the cutaneous-subcutaneous region behind the head of the fibula (Figure 5).

A tablet of calcium (500 mg) once daily was added. She was given counseling for a healthy and hygienic lifestyle with treatment adherence. She continued her treatment and completed the total duration of 24 months. However, a last radiograph of the left knee was unavailable, as she never reported post-completion after 24 months.

## Discussion

Tuberculosis is an outcome of the aerosol inhalation of infected *Mycobacterium tuberculosis* [6]. After the year 1990, there was a surge in cases of drug-resistant tuberculosis, which has threatened public health programs [7].

Skeletal tuberculosis constitutes nearly 3% of all tuberculosis cases [8]. Further, this accounts for about 10-35% of all extrapulmonary tuberculosis cases [8]. It is mostly seen in the spine (40%), hip (25%), and knee (8%) [8]. Primary drug resistance in the absence of any previous history is seldom reported [9]. The involvement of an extrapulmonary site like the knee joint with a rifampicin mono-resistant strain of bacteria is very rare. A detailed search of the literature revealed that no such case has ever been reported in a female where the primary disease was reported with no history of tuberculosis in the past or trauma.

The diagnosis is difficult in such cases mainly due to the fact that the clinical and radiological features lack specificity; often there is an absence of constitutional symptoms of tuberculosis; a lack of awareness and training of primary care physicians results in diagnostic delay; and the paucibacillary nature of the disease [10].

Usually, tests like chest radiography, the Mantoux test, and the interferon gamma-release test have a reasonable level of sensitivity, which could help clinicians [10]. But the isolation of acid-fast bacilli by Ziehl and Neelsen stain on microscopy and culture is the gold standard for treatment initiation [10]. Nevertheless, there is no growth of *Mycobacterium tuberculosis* on culture, and the paucibacillary nature of the disease results in a misdiagnosis or delayed diagnosis [10]. Histopathology (sensitivity 80%) and the GeneXpert, or cartridge-based nucleic acid amplification test, play a significant role in establishing the diagnosis [10].

The mainstay of management is conservative, with anti-tubercular chemotherapy [10]. There are documented algorithms in the national programs available as guidelines for the management of such cases [5]. Surgical management is rarely indicated in cases where there is extensive disease or a chance of disease reactivation [10].

A case similar to the present case was reported by Nithin et al. (2018), where a 26-year-old male was diagnosed with multidrug-resistant tuberculosis [11]. The present case differs from theirs in gender, absence of constitutional symptoms like fever, no history of falls, and tuberculosis [11]. However, their case lacked credibility as he had only rifampicin resistance. Still, they termed it a multidrug-resistant case; there were no images in this report, and this was published in an obscure journal.

A further diagnostic delay could result in severe complications [10]. Therefore, it is essential that all these cases be initiated promptly based on the drug-susceptibility testing. There is a paucity of data related to this condition. However, this was only one case, and data from large centers might help in modifying the existing guidelines for a more targeted approach to rifampicin-resistant knee joint tuberculosis.

## Conclusions

A case of a 26-year-old Indian female is presented who came with left knee swelling, pain, and discharging sinuses. In the absence of a history of tuberculosis or trauma, its diagnosis was an uphill task. However, she was diagnosed with histopathological examination, a cartridge-based nucleic acid amplification test, and radiometric techniques. She underwent a pretreatment evaluation per the national guidelines and was initiated on anti-tubercular chemotherapy. There was some improvement at the six-month follow-up; however, she completed her full course of 24 months. It is essential that there be emphasis on the dissemination of information related to such clinical presentations, as ignorance and a lack of training could result in unfavorable or fatal outcomes.
